# The contribution of physics to Nuclear Medicine: physicians’ perspective on future directions

**DOI:** 10.1186/2197-7364-1-5

**Published:** 2014-05-01

**Authors:** David A Mankoff, Daniel A Pryma

**Affiliations:** Division of Nuclear Medicine, Hospital of the University of Pennsylvania, University of Pennsylvania, 116 Donner Building, 3400 Spruce Street, Philadelphia, PA 19104-4283 USA

**Keywords:** Nuclear Medicine, Physics, Molecular imaging, Future directions

## Abstract

**Background:**

Advances in Nuclear Medicine physics enabled the specialty of Nuclear Medicine and directed research in other aspects of radiotracer imaging, ultimately leading to Nuclear Medicine’s emergence as an important component of current medical practice.

**Discussion:**

Nuclear Medicine’s unique ability to characterize *in vivo* biology without perturbing it will assure its ongoing role in a practice of medicine increasingly driven by molecular biology. However, in the future, it is likely that advances in molecular biology and radiopharmaceutical chemistry will increasingly direct future developments in Nuclear Medicine physics, rather than relying on physics as the primary driver of advances in Nuclear Medicine.

**Summary:**

Working hand-in-hand with clinicians, chemists, and biologists, Nuclear Medicine physicists can greatly enhance the specialty by creating more sensitive and robust imaging devices, by enabling more facile and sophisticated image analysis to yield quantitative measures of regional *in vivo* biology, and by combining the strengths of radiotracer imaging with other imaging modalities in hybrid devices, with the overall goal to enhance Nuclear Medicine’s ability to characterize regional *in vivo* biology.

## Background

From a historical perspective, it can be said that Nuclear Medicine was the direct result of advances in nuclear physics and their translation to medicine. In many ways, physics drove the creation of the field, including our abilities to produce radioisotopes suitable for human use, to capture quantitative data and/or images made possible by these radioisotopes, and to estimate the quantity of radioactive material that could be safely and effectively administered to patients for diagnosis and treatment. Advances in the physics of radioisotope production and radiation detection drove many other aspects of Nuclear Medicine research and development - including the type of radiopharmaceuticals used (^99m^Tc and positron-emitting tracers), the approaches developed for image acquisition and analysis (dynamic imaging, multi-tracer studies, quantitative imaging), and the ability to treat disease using radionuclides (quantitative biodistribution data, dosimetry) - resulting in a confluence of developments that led to major steps forward for the specialty. Physics advances helped create Nuclear Medicine and brought the field to the forefront of medicine as a specialty driven by physical science and technical innovation.

As we look to the future, the tables will be turned somewhat. It is likely that advances in molecular biology and precision medicine will drive clinical care [1]. A heightened understanding of the molecular basis of disease will raise new questions and new demands on imaging to answer those questions. This will, in turn, drive physics advances in Nuclear Medicine. Nuclear Medicine is no longer defined by the physical nature of its imaging probes and therapeutic compounds, but rather by its molecular capabilities. The Society of Nuclear Medicine and Molecular Imaging defines molecular imaging as ‘… the visualization, characterization, and measurement of biological processes at the molecular and cellular levels in humans and other living systems’ [2]. This definition emphasizes the molecular and biologic nature of the imaging, rather than its physical nature. The greatest advantage of radionuclide imaging over other imaging modalities for molecular imaging lies in its ability to characterize and quantify *in vivo* biologic processes at the molecular level without perturbing them. This capability defines the essence of Nuclear Medicine and assures its place in the future of molecular medicine, and it will drive the physics needs of the specialty in the future.

## Discussion

### What are the medical needs that will drive Nuclear Medicine physics advances?

#### Molecularly specific imaging probes to measure regional molecular biology

Increasing sophistication in our ability to characterize genomics and gene expression will require advances in our ability to measure *in vivo* molecular processes using highly sensitive and specific imaging probes. The most sensitive molecular processes - for example, enzyme and receptor biochemistry [3, 4] - operate at submicromolar physiologic conditions, often as low as the nanomolar or picomolar range. Radioisotope imaging is the only current modality that can generate detailed, quantitative human images in this concentration range without impacting the biologic processes under study. The combination of sensitivity and spatial resolution needed to characterize the regional distribution of molecular radiotracers at or below nanomolar concentrations will be a key driver of Nuclear Medicine technology. Chemistry advances that generate highly specific probes will increasingly drive future physics needs, especially with respect to the need for imaging devices with high system sensitivity and quantitative accuracy. Implicit in this consideration is the fact that as more and more specific targets are imaged, less and less output signal will be available.

#### Quantitative biomarker imaging to characterize disease and direct medical treatment

The rapidly increasing array of disease-specific treatments creates a need for methods to characterize disease severity (prognosis), predict response to specific therapies (prediction), and to measure the efficacy of treatment (response). Thus, the focus of radiopharmaceutical imaging will increasingly move away from detection and diagnosis towards characterization. Biomarkers, namely biologic measures that characterize disease status or predict disease behavior [1, 5], play an ever increasingly important role in directing highly tailored, individualized treatment [1]. Nuclear Medicine’s ability to quantify regional *in vivo* biology underlies its unique strength as a method for measuring quantitative *in vivo* biomarkers, thereby providing a unique and much-needed tool for precision medicine [6, 7]. This need will drive research leading to improvements in image quantification and to more sophisticated image analysis. Nuclear Medicine needs to move beyond qualitative or semi-quantitative images of tracer uptake to images of quantitative regional molecular biology - for example, the rate of flux along a specific biochemical pathway [8].

#### Minimizing radiation exposure in diagnostic imaging

There is increasing concern about the risk of low-level radiation exposure arising from diagnostic imaging [9]. This is fueled by the ever-increasing use of imaging in clinical care and concerns that the risk of inducing cancer with diagnostic radiation will counterbalance the benefits of imaging, especially as patients survive previously lethal diseases that make heavy use of imaging such as cardiovascular disease and cancer. The availability of methods such as ultrasound, magnetic resonance imaging (MRI), and optical imaging that do not require ionizing radiation augments the pressure to reduce radiation exposure from diagnostic imaging. These concerns will drive a continued need for getting more and more information from the smallest possible quantities of radiopharmaceutical and for minimizing the radiation arising from associated tests such as the computed tomography (CT) component of positron emission tomography (PET)/CT and single photon emission computed tomography (SPECT)/CT. Methods that afford simultaneous transmission and emission scanning by taking advantage of time-of-flight information [10], for example, may permit lower radiation doses and shorter imaging times. Alternative approaches such as PET/MRI [11] or reconstruction methods that jointly estimate emission and attenuation [12] may eliminate altogether the need for additional radiation exposure to estimate photon attenuation.

#### The increasing importance of radionuclide therapy, including compounds using alpha emitters

Radionuclide therapy already plays an important role in the treatment of several diseases, including hyperthyroidism and thyroid cancer. The unqualified success of Ra-223 dichloride as a therapeutic radionuclide capable of palliating bone pain and extending survival in castrate-resistant prostate cancer [13] suggests that radionuclide therapy using alpha emitters - a highly potent and localized form of radiation - can be effective in ways that beta emitters cannot [14]. This finding, coupled with our increasing ability to create highly specific molecular carriers for radionuclide therapy, suggests a bright future for therapy with alpha emitters and other highly localized radionuclide therapy agents. However, with increasing potency and localization in radionuclide therapy comes the need for increased certainty about the quantitative biodistribution of the therapeutic radiopharmaceutical as well as more sophisticated methods for understanding and quantifying radiation effects at a microscopic level, i.e., radiation microdosimetry [15].

### How will medical needs drive advances in Nuclear Medicine physics?

#### Radionuclide imaging devices need to be more sensitive

The need to reduce radiation exposure, to accurately quantify regional imaging probe biodistribution, and to maximize the molecular information derived from imaging will require improvements in device sensitivity. Most current imaging research has been focused on detectability, i.e., simply visualizing a finding above background. The evolution towards accurate quantification of dynamic processes requires the consideration of not only sensitivity but also temporal resolution. An example is tumor receptor imaging, where target expression may be limited, and the quantity of tracer bound to target will be less than tracers like FDG that are enzymatically trapped [4]. Improvements in image acquisition that reduce imaging uncertainty at low probe concentrations, for example, improved timing resolution for time-of-flight imaging in PET, will be important [16]. Increasingly sophisticated and more robust detection systems will decrease instrumental uncertainty in the location and timing of detected events and improve the quality of information that can be obtained from nuclear imaging probes. Tomograph designs that more fully encompass patient emissions to increase sensitivity may also be important for some applications, especially those that require whole-body distribution measures, for example, radiation dosimetry [17]. Increasingly sophisticated image reconstruction methods that extract the most possible information from each detected radiation event will also contribute to the goal of improved sensitivity [18]. More sophisticated and efficient reconstruction algorithms that consider the time course of tracer concentration [19] and the biologic constraints of the imaged radiopharmaceutical [20, 21] may be particularly helpful in this regard.

#### Radionuclide imaging needs to be quantitative and reproducible

The need for analytic validity of quantitative nuclear imaging measurements [5, 22] - namely accuracy and reproducibility - will direct advances in image generation. This will include advances in instrumentation and operating procedures designed to support devices that are quantitatively consistent from day to day and from patient to patient. In addition, image acquisition and especially image analysis will need to take advantage of information arising from the time course of uptake to create more robust and molecularly accurate measures of regional biology [23]. Higher dynamic range in count rate, enabled for example by detector designs using silicon photomultipliers coupled to individual crystals [24], will be important for applications that require capturing an arterial input function, such as kinetic modeling. Increasing digital storage capacity and processing speed will enable further development of list-mode acquisition, image reconstruction, and image processing that can support this goal.

#### Better image and data analysis methods will make it easier for physicians to get the information they need to direct precision medicine

Advances in computing and the science of molecular imaging analysis will lead to images more relevant to the needs of molecular medicine. Images of regional radiotracer uptake should be accompanied by images of key quantitative molecular parameters, made possible by powerful and user-friendly image analysis software [25]. This software, furthermore, must interface with medical informatics systems to allow the dissemination of these data to the medical care team. Better methods for obtaining parametric images of tracer kinetics will support a broader range of Nuclear Medicine biomarkers in clinical medicine. Improved software for estimating regional radiation dosimetry from quantitative time-varying biodistribution data will support advances in radionuclide therapy. Advances in methods for analyzing ‘big data’ that combine increasingly sophisticated molecular imaging data with data on tissue genomics and gene expression obtained from tissue sampling will better able to direct individualized medicine.

#### Combining information derived from other imaging modalities with radiotracer-based molecular imaging will improve our ability to characterize complex *in vivo* molecular biology in patients

The introduction of hybrid imaging in the form for PET/CT and SPECT/CT has changed the way Nuclear Medicine is practiced by offering the ability to directly relate regional radiotracer uptake to structural anatomy [26]. More recently, PET/MRI hybrid devices have been introduced, with early clinical applications driven largely by MRI’s ability to define anatomy in locations such as the brain and pelvis [11]. Hybrid imaging will continue to play an important role in Nuclear Medicine, but the future of Nuclear Medicine as a molecular specialty depends upon the ability to move beyond the marriage of anatomy and tracer distribution to more integrated approaches for quantitative molecular imaging. Refinements in instrumentation (both in nuclear and non-nuclear modalities) and improvements in image analysis should allow us to combine the strengths of the different modalities to better measure *in vivo* biology. For example, the combination of hyperpolarized ^13^C probes [27] and nuclear probes enabled by PET/MRI should allow us to tackle measurements not possible by either method alone, for example, characterization of tumor energy metabolism and the relative use of metabolic substrates such as glucose, lactate, and glutamine [28]. Other modalities such as optical imaging and MEG may also be possible. These needs will drive further integration of hybrid imaging modalities not only for image acquisition, but also equally importantly for image analysis.

## Summary

The increasing importance of molecular medicine and Nuclear Medicine’s unique ability to quantify regional *in vivo* molecular biology assure Nuclear Medicine an ongoing and significant role in medical science and the practice of medicine. Whereas advances in physics drove the early creation and evolution of the field of Nuclear Medicine, future developments will be increasingly guided by advances in molecular biology and molecular imaging probe chemistry. In turn, advances in the specialty supported by Nuclear Medicine physics will lead to advances in Nuclear Medicine’s ability to measure regional *in vivo* biology as an essential component of current and future medical care.
